# Patients with migraine with aura have increased flow mediated dilation

**DOI:** 10.1186/1471-2377-10-18

**Published:** 2010-03-10

**Authors:** Fabrizio Vernieri, Leo Moro, Claudia Altamura, Paola Palazzo, Raffaele Antonelli Incalzi, Paolo Maria Rossini, Claudio Pedone

**Affiliations:** 1 Neurologia Clinica, Università Campus Bio-Medico, Roma, Italy; 2 Associazione Fatebenefratelli per la Ricerca AFaR, Dipartimento di Neuroscienze, Ospedale 'San Giovanni Calibita' Fatebenefratelli, Isola Tiberina, Roma, Italy; 3 Cattedra di Geriatria, Università Campus Bio-Medico, Roma, Italy

## Abstract

**Background:**

Endothelium-derived nitric oxide (NO) mediates the arterial dilation following a flow increase (i.e. flow-mediated dilation, FMD), easily assessed in the brachial artery. NO is also involved in cerebral hemodynamics and it is supposed to trigger vascular changes occurring during migraine. This study aimed at investigating whether migraine patients present an altered response to NO also in the peripheral artery system.

**Methods:**

We enrolled 21 migraineurs (10 with aura [MwA], 11 without aura [MwoA]), and 13 controls. FMD was evaluated with ultrasound in all subjects by measuring the percentage increase of the brachial artery diameter induced by hyperaemia reactive to sustained cuff inflation around the arm above systolic pressure. FMD values were then normalized for shear stress.

**Results:**

Normalized FMD values were higher in patients with MwA (28.5 10^-2^%.s) than in controls (9.0 10^-2^%.s) and patients with MwoA (13.7 10^-2^%.s) (p < 0.001). FMD was over the median value (19%) in 23.1% of controls, in 45.5% of the MwoA patients, and in 90% of the MwA patients.

**Conclusions:**

Migraineurs with aura present an excessive arterial response to hyperaemia, likely as an effect of an increased sensitivity to endothelium-derived nitric oxide. This phenomenon observed peripherally might reflect similar characteristics in the cerebral circulation.

## Background

Migraine is a common disabling primary headache. Over the past decades, several neuronal and vascular hypotheses have been proposed to clarify its pathophysiology [1]. Today, migraine is considered a neurovascular disorder and classified into migraine without aura (MwoA) and migraine with aura (MwA); the latter is usually preceded, or sometimes accompanied, by focal neurological symptoms [2].

The dilatation of the intracranial vessels, part of the trigemino-vascular system, is a well-known vascular feature of migraine attacks [3]. It is thought to be part of a basic pain-sensitizing process engaging the nitric oxide (NO) cascade. In fact, NO can trigger migraine pain [4] and its liberation, due to glyceryl trinitrate administration, induces a more pronounced intracranial arterial dilation in migraineurs than non-migraineurs [5]. Besides, some antimigraine drugs are supposed to act through the inhibition of NO and of the cascade of intracellular reactions triggered by NO [5].

Endothelium-derived NO is also considered the principal mediator of flow-mediated dilation (FMD), a phenomenon characterized by artery dilatation in response to a flow increase [6-8]. Although FMD precise mechanism is not completely understood, the shear stress on the vessel walls determined by a flow increase is supposed to activate ion channels and the endothelial NO synthase, with the subsequent generation of NO and therefore a vasodilatation. FMD thus reflects the arterial capability to self-regulate its tone in response to changes in the local environment (i.e. physical and chemical stimuli in the lumen). FMD is easily assessed in the brachial artery, but it is considered a marker of endothelial function for the entire vascular tree [8-10]. Moreover, NO dependent vasodilatation is also involved in the chemoregulatory mechanisms subtending cerebral vasomotor reactivity (VMR) to hypercapnia [10]. Patients with MwA were reported to have a cerebral vasomotor reactivity to CO_2 _inhalation higher than MwoA patients and controls [11-13].

On the other hand, the possible role of NO and endothelial dysfunction in patients affected by migraine was explored so far in few studies with negative results [14,15]. Nevertheless, the authors of these studies concluded that a defined conclusion on this issue could not be drawn.

The present study aimed to investigate whether the vascular response to endothelium-derived NO differs in controls, in patients with migraine without aura and with migraine with aura.

### Subjects

Twenty-one patients affected by migraine, 11 without aura and 10 with aura, diagnosed according to IHS criteria [2], and 13 healthy subjects were enrolled in the study. At the time of examination, all included patients were headache-free and during a migraine-drug withdrawal period of one week; all patients had discontinued prophylactic medications for at least one month before the study. All enrolled subjects underwent neurological examination and endothelial function assessment, after detailing past medical history and drug consumption.

## Methods

We evaluated endothelial function by measuring the change in forearm blood flow induced by flow mediated dilation [16,17]; measurements were performed according to guidelines [18].

All examinations were performed by a single experienced vascular sonographer, who was unaware of the subjects' clinical background, using an ultrasound system (Aplio 80 CV, Toshiba) with a broadband 8-14 MHz transducer. In our lab coefficient of variation for FMD repeated measurements is 15%.

Because of circadian variations of peripheral vascular tone, the FMD investigation was performed on all patients between 8 and 9 AM in a quiet, temperature controlled room (22°C to 24°C). All subjects were studied after a 12-hour overnight fast. Smokers refrained from smoking in the 12 hours preceding the study. Vasoactive drugs were discontinued in the same time period. Female subjects were investigated during the first week of menstrual cycle [19]. Patients were headache-free for at least 15 days at the moment of the examination. No patient had a migraine attack in the five days subsequent the examination.

**Figure 1:**
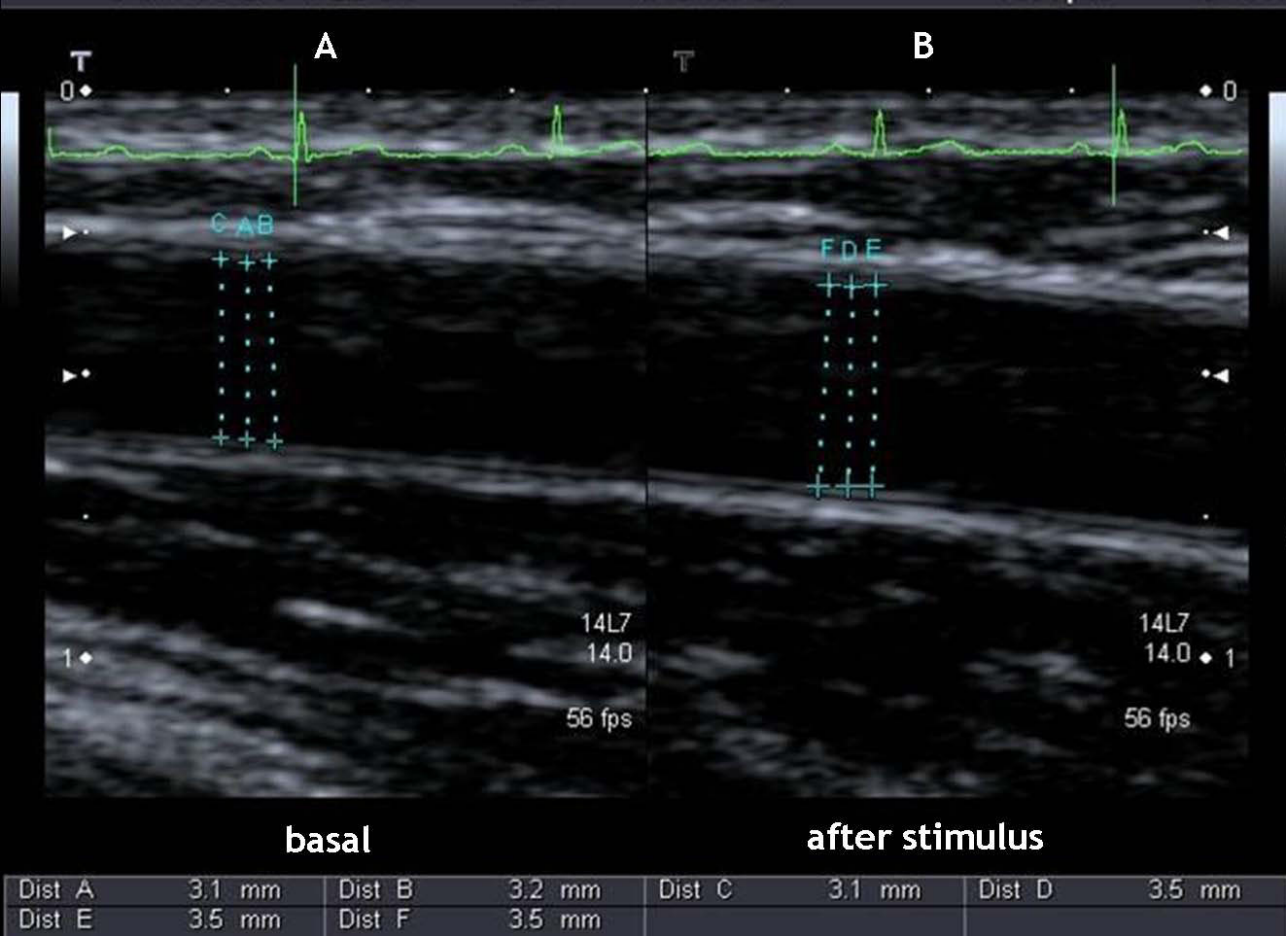
**Ultrasound image of the brachial artery at (A) baseline and (B) 60 seconds after hyperaemic stimulus**.

With the patient in the supine position, the right brachial artery was scanned over a longitudinal section, 3-5 cm above the elbow. Depth and gain settings were optimized to identify the lumen-to-vessel wall interface. The FMD was assessed by measuring the change in brachial artery diameter after 50, 60 and 70 seconds of reactive hyperemia, compared with baseline measurements, after deflation of a cuff placed around the forearm that had been inflated to 50 mm Hg above systolic blood pressure for 5 minutes (figure 1). Arterial diameter was determined as the internal dimension of the vessel wall from the anterior-to-posterior interface between the lumen and the intima. The mean diameter was calculated from three measurements of arterial diameter performed at end-diastole incident with the R wave on a continuously recorded ECG.

The response of the vessel diameter to reactive hyperemia (FMD) was expressed as a percent change relative to the diameter before cuff inflation. However, FMD depends on the shear stress on the blood vessels, which is directly related to the velocity and the viscosity of the blood but inversely related to the vessel diameter. Vessels with different diameters may have the same flow but substantially different levels of shear stress and thus a different degree of stimuli for FMD. In other words, FMD values derived from subjects with a comparable endothelial function but with different vessel diameters may results dissimilar. To avoid this bias, FMD raw values need to be corrected for flow velocity and diameter. A shear rate was then estimated as velocity divided by diameter [20]. Peak shear rate, estimated as peak flow velocity divided by baseline diameter, was calculated to quantify the FMD stimulus in each subject. FMD responses were normalized by dividing the maximal percentage change in diameter by the peak shear rate [20].

The experimental protocol was approved by the University Ethical Committee, and all patients and controls signed a written informed consent.

### Analytic approach

To describe the characteristics of our populations we used the mean with inherent standard deviation and proportions for continuous and discrete variables, respectively.

To compare the relationship between age and FMD we used the Spearman's correlation coefficient, while to compare the mean FMD between gender and across the three groups (controls, MwoA, and MwA) we used a generalized linear model (GLM) with Tukey's correction for multiple comparisons and the median test. The GLM approach was also used to evaluate differences of the FMD in these groups taking into account age and gender, and to calculate adjusted means of the FMD using the least-squares method.

## Results

Overall, we evaluated 34 participants: 21 with migraine (10 with aura [MwA], 11 without aura [MwoA]), and 13 controls. Mean age of the sample was 36.7 years (SD: 10), without differences between MwA and MwoA (41.2 and 40.1 years, respectively), while controls were younger (mean age: 30.5 years). The majority of participants were women (28/34, 82%), with higher prevalence in the MwoA group (10/11, 91%). Only four participants (3 in in the group MwoA, one control) were active smokers, three (2 MwoA, 1 MwA) had hypertension, and two participants in MwA group had dyslipidemia. None of the participants had a diagnosis of diabetes mellitus.

As shown in table 1, basal systolic blood flow velocity was higher in the control group compared to MwA group (P < 0.05), but not compared to the MwoA group. Post-ischemic systolic flow velocity and shear rate showed a similar pattern, with lower values observed in the MwA group compared to the control group. Overall mean FMD was 20.9% (95% CI: 17.0% - 24.8%), with figures of 15.5% in controls, 18.2% in the MwoA group, and 31.0% in the MwA group (P < 0.05 vs. both controls and MwoA). The median test confirmed the result (P < 0.001).

**Table 1:** Comparison of hemodynamic variables

	Controls Mean (95% CI)	Migraine without aura Mean (95% CI)	Migraine with aura Mean (95% CI)
**Basal systolic blood flow velocity (cm.s**^-1^**)**	32.2 (25.9 - 38.4)	27.0 (20.2 - 33.8)	22.0 (16.2 - 27.8)*
**Post-ischemic flow velocity (cm.s**^-1^**)**	47.0 (41.2 - 52.7)	41.3 (33.4 - 49.1)	34.6 (24.8 - 44.3)*
**Baseline artery diameter (mm)^§^**	2.74 (2.42 - 3.06)	3.15 (2.76 - 3.53)	3.13 (2.64 - 3.62)
**Shear rate (s**^-1^**)**	177.0 (148.9 - 205.1)	134.6 (104.7 - 164.5)	119.2 (71.5 - 167.0)*
**Flow mediated dilation (% of basal)**	15.5 (13.3 - 17.6)	18.2 (14.1 - 22.3)	31.0 (21.7 - 40.3)*,^†^

* P < 0.05 vs. controls

† P < 0.05 vs. MwoA

§Mean of 3 measurements

FMD was not correlated with age (Spearman's correlation coefficient: 0.118; P = 0.5), while women tended to have higher mean FMD compared to men (respectively: 22.1%, 95% CI: 17.4% - 26.7%; 16.3%, 95% CI: 11.4% - 21.2%; P = 0.30). Results from a multivariable generalized linear model adjusted for age and gender are similar to the one obtained in the unadjusted analysis: the adjusted means of FMD were 12.0% (95% CI: 6.0% - 18.0%) in the control group, 16.3% (95% CI: 9.7% - 23.0%) in the MwoA group, and 30.7% (95% CI: 23.9% - 37.5%) in the MwA group (P < 0.01 vs. both controls and MwoA). Age and gender were not associated with FMD in this model (P = 0.108 and P = 0.145, respectively).

**Figure 2:**
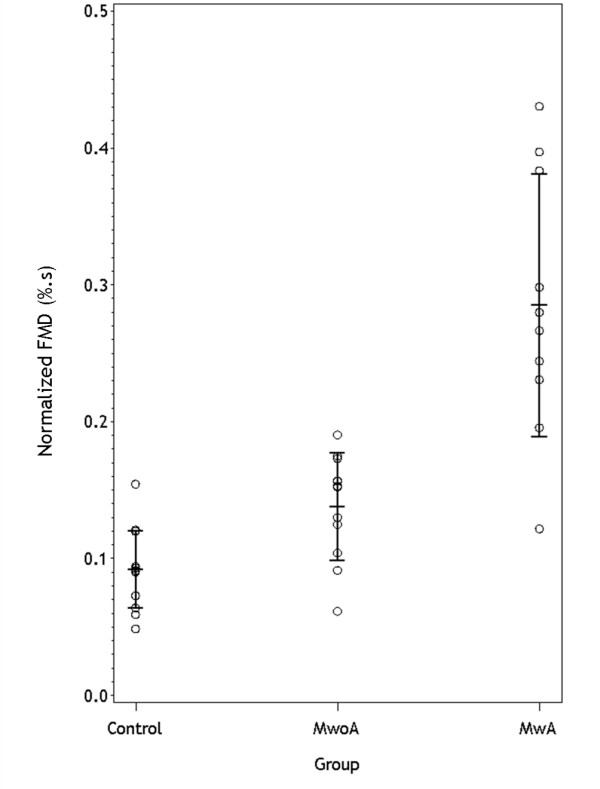
**FMD normalized for the peak shear rate in the three groups considered (controls, migraine without aura [MwoA], migraine with aura [MwA])**. Circles: individual values; middle horizontal tick: mean; bars: ± 1 SD.

The results did not change when we analyzed the FMD normalized for the peak shear rate (figure 2): it was 9.2 10^-2 ^%.s (95% CI: 7.5 - 10.9) in the control group, 13.7 10^-2 ^%.s (95% CI: 11.1 - 16.4) in the MwoA group and 28.5 10^-2 ^%.s in the MwA group (95% CI: 21.6 - 35.4) (P < 0.001). The corresponding least-squares means adjusted for age and gender were 7.7 10^-2 ^%.s (95% CI: 4.0 - 11.5), 15.0 10^-2 ^%.s (95% CI: 10.7 - 19.2), and 30.5 10^-2 ^%.s (95% CI: 26.1 -34.8) (P < 0.001).

The proportion of subjects with FMD over the median value (19%) was 23.1% in the control group, 45.5% in the MwoA group (OR vs. control: 2.78; 95% CI: 0.48 - 16.03), and 90% in the MwA group (OR vs. control: 30.0; 95% CI: 2.62 - 342.73).

## Discussion and Conclusions

The main finding of the present study is that patients affected by migraine with aura show a higher FMD than controls and migraineurs without aura. In addition, our data suggest that FMD values range with continuity from controls to patients with MwoA to those with MwA.

Flow mediated dilation is a physiological phenomenon characterized by vessel dilatation in response to an increase in blood flow, or more precisely to the shear stress, a mechanical stimulus resulting from blood stream along the endothelial cells [20]. An important mediator of FMD is endothelium-derived NO [6-8]. Hyperemic stimuli induce the endothelium to release NO which acts directly on arterial smooth muscle cells producing vasodilatation. As mentioned before, NO is a potent endogenous vasodilator, involved in pain transmission, hyperalgesia, chronic pain, inflammation and central sensitisation. It is also involved in migraine pathogenesis causing an immediate headache in all migraine patients and less often in healthy subjects [21-23]. In addition, NO is released during cortical spreading depression (CSD), playing a significant role in the hemodynamic changes associated with this phenomenon [24-27].

Migraine patients seem to have an arterial super-sensitivity to NO [5,28]. NO-containing nerve fibers have been demonstrated around intracranial arteries suggesting that NO probably plays a regulatory role in neuropeptide release (e.g. calcitonin gene-related peptide, CGRP) [29,30], starting a neurogenic inflammatory process. Consequently, an increased response to NO might not only imply an excessive vasodilation by the direct action of NO on the vessel wall, but also an excessive release of CGRP from perivascular nerves induced by NO.

Arterial super-sensitivity to NO may be explained also by an autonomic dysfunction. Some authors [3,31] reported a possible impairment of parasympathetic and, in particular, sympathetic control of cerebral blood flow in migraine patients. Muller and Marziniak [32] speculated that this autonomic dysfunction could subtend the disorder of cerebral autoregulation demonstrated in migraine patients also by previous studies [33,34]. Lacking a functional autonomic controls, cerebral vessels would become over-sensible to NO as well as to other chemical stimuli, such as CO_2_. This hypothesis is sustained by several studies describing an increased cerebrovascular reactivity to hypercapnia in migraine patients, particularly in those affected by MwA [10-13]. Thus, our findings are in line with those reported on the cerebral circulation, supporting the theory that patients affected by MwA present a substantial disorder of autoregulation inducing a super-sensitivity to chemical stimuli (e.g. NO, CO_2_).

Previous studies have already investigated the endothelial function and NO vascular response describing either a normal (14, 15, 35) or a reduced FMD [36,37] in migraine patients respect to controls. However, two studies evaluated only patients affected by MwoA [35,36], and two studies did not distinguish between MwA and MwoA patients in their analytic approach [14,37]. Only one study analysed the FMD normalized for the peak shear rate [37], in order to correct FMD values for baseline diameter and peak flow velocity. In our study MwA patients show a higher FMD than controls and MwoA subjects both in terms of percentage increase in diameter and of values normalized for peak shear stress. Controls tended to have higher shear rate compared to migraneurs, and this is expected given the fact that migraneurs have slightly greater artery diameter. Correction for this variable, however, did not change our results.

Differently from all these previous studies that were carried out by means of an ultrasound probe with a frequency of 7.5 -10 MHz, we performed our examination with a higher frequency probe (14 MHz). In our opinion, this issue is of remarkable importance since higher frequency probes have greater imaging resolution when evaluating superficial vessels. In our case, the brachial artery walls are located within few millimetres from skin surface. In this situation a probe with a higher frequency allows for a more precise detection of minimal calliper variation, while the intima layer of the superficial wall of the artery can not be accurately visualized with a lower frequency probe, resulting in a less accurate measure.

Silva et al. [15] assessed also blood levels of fasting nitrates and nitrites in controls and patients affected by MwA or MwoA during the asymptomatic period, finding no differences in the three groups. Fasting nitrates and nitrites were measured before FMD evaluation, thus these findings do not exclude a higher release of NO in migraine patients in response to a shear stress. Moreover, these data differ from those of D'Amico et al. [38] who described a hyperactivity of L-arginine-NO pathway.

However, these conflicting results do not invalidate the interpretation of our findings. In fact, a high FMD in patients with MwA could be the result of either an increased release of NO induced by shear stress (nearly impossible to measure locally) or of an arterial super-sensitivity to NO. We favour the latter explanation, as it is also in agreement with the evidence that MwA patients' cerebral arteries present a hyper-reactivity to chemical stimuli [11-13]. We can speculate that MwA patients' arteries are peculiarly characterized by a super-sensitivity to chemical and physical stimuli throughout the whole arterial system.

We are aware that the present study has some limitations such as the small samples and the younger age in controls (30.5 years) compared with patients (40.1 and 41.2 years). Another flaw of our study is that we cannot raise any definite conclusion concerning a possible increased sensitivity to endothelium-derived NO in absence of a control experiment with an endothelium-independent NO donor, namely GTN, which we were not able to perform in our study setting.

Further studies are needed to verify if this super-sensitivity plays a key role in determining the aura phenomenon and represents a risk condition for vascular events. Moreover, a study investigating a larger population would be necessary to confirm a continuum of endothelial reactivity (i.e. FMD values) from controls to migraine without aura to migraine with aura.

## Competing interests

The authors declare that they have no competing interests.

## Authors' contributions

FV made substantial contribution to conception and design of the study and to analysis and interpretation of data; LM was our experienced vascular sonographer, who carried out all the FMD measurements by ultrasound system; CA made substantial contributions to analysis and interpretation of data and was involved in drafting the manuscript and revising it critically; PP examined and enrolled all participants of the study; RAI and PMR revised the paper critically for important intellectual content and gave final approval of the version to be published; CP made substantial contributions to analysis and interpretation of data and was involved in drafting the manuscript. All authors read and approved the final manuscript.

## Pre-publication history

The pre-publication history for this paper can be accessed here:

http://www.biomedcentral.com/1471-2377/10/18/prepub
